# Fatal antiphospholipid syndrome following endoscopic transnasal-transsphenoidal surgery for a pituitary tumor: A case report

**DOI:** 10.1097/MD.0000000000005774

**Published:** 2017-01-10

**Authors:** Chiao-Zhu Li, Chiao-Ching Li, Chih-Chuan Hsieh, Meng-Chi Lin, Dueng-Yuan Hueng, Feng-Chen Liu, Yuan-Hao Chen

**Affiliations:** a Department of Neurological Surgery, Tri-Service General Hospital, National Defense Medical Center, Taipei; b Department of Surgery, Kaohsiung Armed Forces General Hospital; c Department of Surgery, Zoying Branch, Kaoshiung Armed Forces General Hospital; d Division of Rheumatology, Immunology, and Allergy, Department of Medicine, Tri-Service General Hospital, National Defense Medical Center, Taipei, Taiwan.

**Keywords:** antiphospholipid syndrome, endoscopic transsphenoidal approach, pituitary microadenoma, plasma exchange, young stroke

## Abstract

**Introduction:**

The fatal type of antiphospholipid syndrome is a rare but life-threating condition. It may be triggered by surgery or infection. Endoscopic transnasal-transsphenoidal surgery is a common procedure for pituitary tumor. We report a catastrophic case of a young woman died of fatal antiphospholipid syndrome following endoscopic transnasal-transsphenoidal surgery.

**Methods and Result:**

A 31-year-old woman of a history of stroke received endoscopic transnasal-transsphenoidal surgery for a pituitary tumor. The whole procedure was smooth. However, the patient suffered from acute delirium on postoperative day 4. Then, her consciousness became comatose state rapidly with dilatation of pupils. Urgent magnetic resonance imaging of brain demonstrated multiple acute lacunar infarcts. The positive antiphosphoipid antibody and severe thrombocytopenia were also noted. Fatal antiphospholipid syndrome was diagnosed. Plasma exchange, corticosteroids, anticoagulant agent were prescribed. The hemodynamic condition was gradually stable. However, the consciousness was still in deep coma. The patient died of organ donation 2 months later.

**Conclusion:**

If patients have a history of cerebral stroke in their early life, such as a young stroke, the APS and higher risk of developing fatal APS after major surgery should be considered. The optimal management of APS remains controversial. The best treatment strategies are only early diagnosis and aggressive therapies combing of anticoagulant, corticosteroid, and plasma exchange. The intravenous immunoglobulin is prescribed for patients with refractory APS.

## 1 Introduction

The antiphospholipid syndrome (APS) is a critical and potentially fatal medical condition. The fatal type of APS (ex. Catastrophic APS; CAPS) is estimated to develop in <1% of all APS patients. Although some precipitating factors having been identified, APS may sometimes occur in the absence of an obvious triggering factor. Here, we report a young woman who presented with pituitary adenoma and developed fatal APS after undergoing an endoscopic transnasal-transsphenoidal surgery to remove a pituitary tumor, complicated with brain infarction.

## 2 Case report

**Figure 1:**
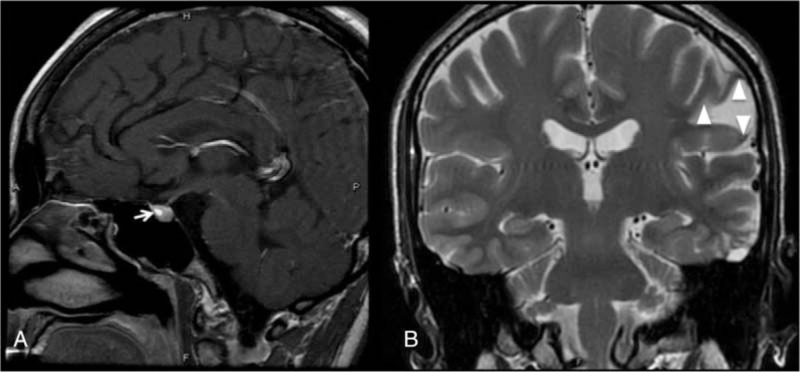
The pituitary microadenoma (arrow) is shown in a sagittal T1-weighted gadolinium contrast-enhanced MRI (A); A coronal, T2-weighted brain magnetic resonance image depicts focal encephalomalacia in the left frontal lobe (arrow head) (B). MRI = magnetic resonance imaging.

A 31-year-old woman with a 3-year history of amenorrhea and galactorrhea was referred to our neurosurgery outpatient department (OPD) 1 year earlier, following a diagnosis of a pituitary microadenoma (Fig. 1A). She sustained a head injury in a traffic accident when she was 19-year-old, after which she began experiencing seizures. In addition to pituiyary microadenoma, magnetic resonance imaging (MRI) of pre-operation also revealed focal encephalomalacia in the left frontal lobe (Fig. 1B) that was thought to be due to her previous head injury. During the 1-year follow-up at our OPD, the tumor diameter increased from 3.5 to 5.0 mm with amenorrhea developing, though her serum prolactin level increased a little bit (from 5.6 to 8.59 μg/dL). Her preoperative blood test results were all within normal ranges. Conservative treatment of an oral dopamine agonist and regular follow-up were suggested initially, but she decided receiving surgical treatment later. Tumor removal via an endoscopic transnasal-transsphenoidal was performed and the sellar region was repaired with fat grafting and Tissuecol Duo (Baxtar, Deerfield, IL) at the end of the procedure.

**Figure 2:**
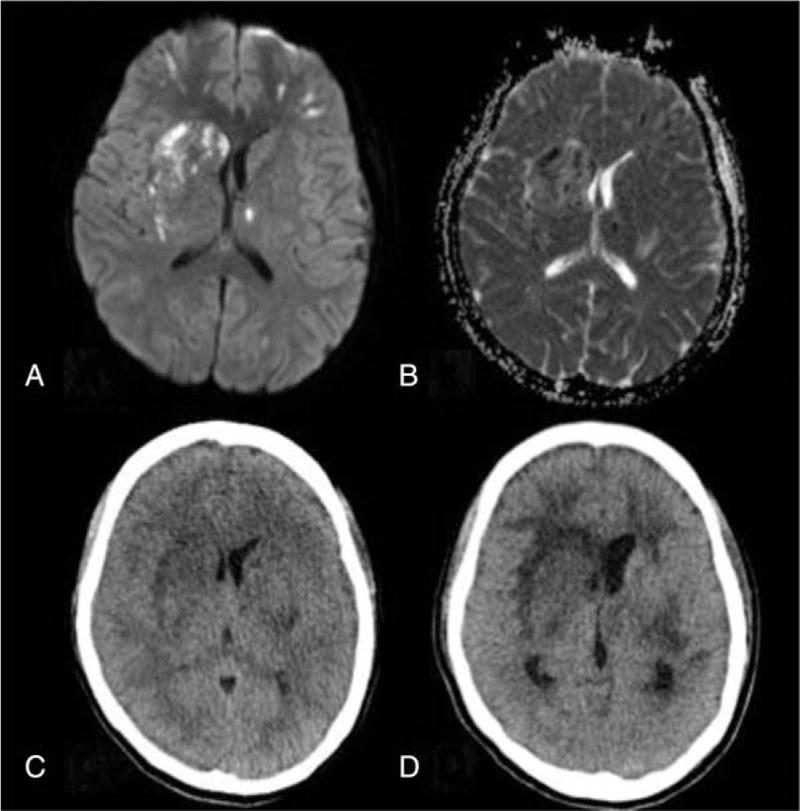
Multiple cerebral infarctions are evident in (A) a diffusion-weighted image, (B) an apparent diffusion coefficient map; brain CT scan showing progressive edema on postoperative days 4 (C) and 7 (D). CT = computed tomography.

On postoperative day 4, acute delirium of a low-grade fever was noted. The routine blood test results revealed leukopenia (WBC: 2140/μL) and thrombocytopenia (platelet count: 84,000/μL). Both C-reactive protein (22.13 mg/dL) and d-dimer level (35.20 mg/L fibrinogen equivalent units) increased. Brain computed tomography (CT) showed hypodensities in the bilateral frontal lobes, left temporal lobe, and left basal ganglion with severe swelling of the right frontal lobe which compressed right lateral ventricle (Fig. 2C). To determinate the extend of infarction area, the urgent brain MRI was performed which confirmed multiple acute lacunar infarcts in the bilateral frontal lobes, left parietal lobe, left occipital lobe, bilateral basal ganglia, and left thalamus (Fig. 2A and B). In susceptibility weighted images, multiple small low-signal intensities, suggestive of petechial hematomas, were identified in the infarcted areas. Her consciousness deteriorated to comatose state and ventilation support was prescribed. Then, leukopenia (2230/μL) and severe thrombocytopenia (46,000/μL) were profound with increment of her activated partial thromboplastin time to 37.1 s. A thrombosis screening showed normal levels of anticardiolipin immunoglobulins G and M; the anti-β2 glycoprotein I (0.6 u/mL) was within normal range, but the level of lupus anticoagulant was high (2.34). Her antithrombin III, protein C, and protein S levels were depressed to 54.4% (normal range, 70–120%), 52.3% (normal range, 70–140%), and 38.6% (normal range, 60–110%), respectively.

Analyses for rheumatic diseases (antinuclear antibody, rheumatoid factor, antidouble-stranded DNA antibody) were unremarkable. Proteinuria was noted with urine dipstick values of 4+. Sepsis was also impressed with positive results in sputum and blood cultures (*Streptococcus pneumonia* and *Acinetobacter baumannii*). Although plasma exchange, corticosteroids, anticoagulant agent, and empiric antibiotic treatment were administered under impression of fatal type of APS, deep coma (Glasgow Coma Scale score E1M1VT) with central failure still occurred at postoperative day 9 while repeat CT scan of brain revealed profound infarctions and diffuse brain swelling (Fig. 2D). Therapeutic plasma exchange of 5 sessions using fresh frozen plasma as replacement fluid was also performed. The vital signs were gradually stable without an inotropic agent. However, the patient was still in comatose status. Two months later, the family decided to donate her organs finally.

## 3 Discussion

To our knowledge, this is the first reported case of fatal CAPS following endoscopic transnasal-transsphenoidal approach tumor resection. The diagnostic criteria of APS include at least 1 clinical criterion (vascular thrombosis and pregnancy morbidity) and 1 laboratory criterion.^[1]^ CAPS is a rare APS subtype, accounting for less than 1% of all patients with APS, and has a mortality rate of 50%.^[2]^ CAPS is characterized by multiple organ dysfunction and failure due to widespread small vessel thrombosis within a short period.^[3,4]^

The *2002 International Congress* described the classification criteria for CAPS to include (1) evidence of the involvement of 3 or more organs, systems, and/or tissues; (2) development of manifestations simultaneously, or within 1 week; (3) histopathological confirmation of small vessel occlusion in at least 1 organ or tissue; and (4) laboratory confirmation of the presence of antiphospholipid antibodies (lupus anticoagulant and/or anticardiolipin antibodies).^[5]^ Our patient exhibited brain infarction, proteinuria, severe thrombocytopenia, respiratory failure, and a positive lupus anticoagulant antibody titer, all of which developed within 1 week, without histopathological confirmation of small vessel occlusion. She was, therefore, diagnosed with fatal APS, possible CAPS. The precipitating factors for CAPS include infection, surgery, anticoagulation withdrawal/low international normalized ratio, medications, obstetric complications, neoplasms, and systemic lupus erythematosus flares.^[6]^ The most common causes of death in patients with CAPS are cerebral events (mainly ischemic stroke) and infection.^[7]^ In our patient, fatal APS might be triggered not only by surgery but also by sepsis.

The fatal type of APS is hard to prevent due to its rarity and the lack of clear precipitating factors. APS may be a “second-hit” syndrome that the antiphospholipid antibody positive patient superimpose with events like surgery or infection.^[8]^ Therefore, the fatal APS should be considered immediately in the patients with following findings: (1) preoperative images indicate young stroke, (2) with/without multiple unexpected abortions, and (3) postoperative small vessel occlusion in at least 1 organ or tissue occurred.

The acceptable recovery rate of treatments for APS is achieved with a combination of anticoagulant, corticosteroid, and plasma exchange (77.8%), followed by above combined therapy with intravenous immunoglobulin (69%).^[9]^ Plasma exchange can remove antiphospholipid antibodies, cytokines, and complement, improving the survival rate. Intravenous immunoglobulin should be considered in APS cases refractory to plasma exchange, especially when a severe infection develops.^[9]^ In our patient, the hemodynamic condition became stable for a long period after therapeutic plasma exchange. However, because of persistent coma, the patient dead of organ donation at last. The APS patients need intensive care and avoid intravascular instrumentation due to high risk of new clot formation.^[10]^

## 4 Conclusion

If patients have a history of cerebral stroke in their early life, such as a young stroke, the APS and higher risk of developing fatal APS after major surgery should be considered. The optimal management of APS remains controversial. The best treatment strategies are only early diagnosis and aggressive therapies combing of anticoagulant, corticosteroid, and plasma exchange. The intravenous immunoglobulin is prescribed for patients with refractory APS.
